# Effects of intravenous immunoglobulin therapy on behavior deficits and functions in sepsis model

**DOI:** 10.1186/s13613-015-0062-z

**Published:** 2015-07-31

**Authors:** Perihan Ergin Ozcan, Evren Senturk, Gunseli Orhun, Salih Gumru, Nadir Arican, Nurcan Orhan, Canan Ugur Yılmaz, Mehmet Kaya, Feyza Aricioglu, Figen Esen

**Affiliations:** Department of Anesthesiology, Istanbul Faculty of Medicine, Istanbul University, Capa-Fatih, 34039 Istanbul, Turkey; Department of Pharmacology and Psychopharmacology Research Unit, Faculty of Pharmacy, Marmara University, Istanbul, Turkey; Department of Forensic Medicine, Istanbul Faculty of Medicine, Istanbul University, Capa-Fatih, 34039 Istanbul, Turkey; Department of Neuroscience, Institute of Experimental Medicine, Istanbul University, Capa-Fatih, 34039 Istanbul, Turkey; Department of Physiology, Istanbul Faculty of Medicine, Istanbul University, Capa-Fatih, 34039 Istanbul, Turkey

**Keywords:** Sepsis, Immunoglobulins, Cecal ligation and puncture, Plus maze test, Forced swimming test, Behavioral alteration

## Abstract

**Background:**

We aim to demonstrate behavioral alterations in a sepsis model using intravenous (IV) immunoglobulin G (IgG) and immunoglobulins enriched with IgA and IgM (IgGAM).

**Methods:**

We divided 48 Wistar albino rats into five groups: control group, sham-operated group (only antibiotic treatment), cecal ligation and puncture (CLP) group (CLP plus antibiotic treatment), IgG group (250 mg/kg IV IgG) and IgGAM group (250 mg/kg IV IgGAM). Intravenous immunoglobulins were given 5 min after the CLP procedure. Experimental animals put into three behavioral tasks 10, 30 and 60 days after the surgery; to evaluate the locomotor activity, an open field test was performed, elevated plus maze test was used to measure anxiety levels, and depressive state was assessed by forced swimming test. The effects of therapy which were acquired from the results of these tests were used to estimate the behavioral changes after CLP.

**Results:**

The mortality rate of 50% in the septic rats decreased to 30 and 20% with the administration of IgG and IgGAM, respectively. Significant changes on locomotor activity and depressive-like behavior were reported in the sepsis group; on the other hand, the treatment with immunoglobulins reduced the symptoms. Treatment with immunoglobulins attenuated the sepsis-related anxiogenic-like responses. Behavioral alterations returned to normal on day 60 in all groups.

**Conclusions:**

Sepsis caused deterioration on behavioral parameters. Immunoglobulin treatments alleviated the symptoms of functional disturbances and caused early reversal of behavioral deficits in septic animals.

## Background

Acute brain dysfunction is a common complication of sepsis, which has been reported to be associated with adverse outcomes like long-term cognitive impairments. Although the underlying mechanism remains poorly understood, inflammation causing cerebral endothelial activation and blood–brain barrier (BBB) alterations were reported in several models of septic brain [1, 2]. Cognitive consequences of sepsis were tested in septic rats in which survivors showed learning and memory impairment after complete physical recovery in a CLP model [3]. Other experimental studies also suggested this model as a clinically relevant method to investigate cognitive impairment in sepsis survivors [4, 5]. Treatments targeted to manipulate the inflammatory pathways have been investigated to influence long-term cognitive deficits in sepsis-induced brain dysfunction. Various therapeutic interventions have been tested in experimental trials. The beneficial effects of antioxidant treatment in long-term memory impairment were reported in a cecal ligation and puncture (CLP) model in rats [4]. Ritter et al. showed antioxidant treatment with *N*-acetylcysteine and deferoxamine prevents cognitive impairment in septic mice [6]. Guanosine treatment was reported to reduce oxidative stress and recovered the impaired memory in CLP-induced septic rats [7]. Administration of epinephrine, naloxone, dexamethasone and glucose in septic survivors reversed long- term cognitive impairment [8]. We recently demonstrated that treatment with intravenous immunoglobulin protects the BBB integrity and inhibits CLP-induced sickness behavior, and improves survival in septic animals [9].

In this study, we want to investigate the time-dependent protective effects of a single dose of standard Immunoglobulin G (IgG) and immunoglobulin’s enriched with IgA and IgM (IgGAM) intravenous (IV) administration on long-term behavioral alterations including depression and anxiety-like behavior in CLP-induced sepsis model in rats.

## Methods

### Animals

**Fig. 1:**
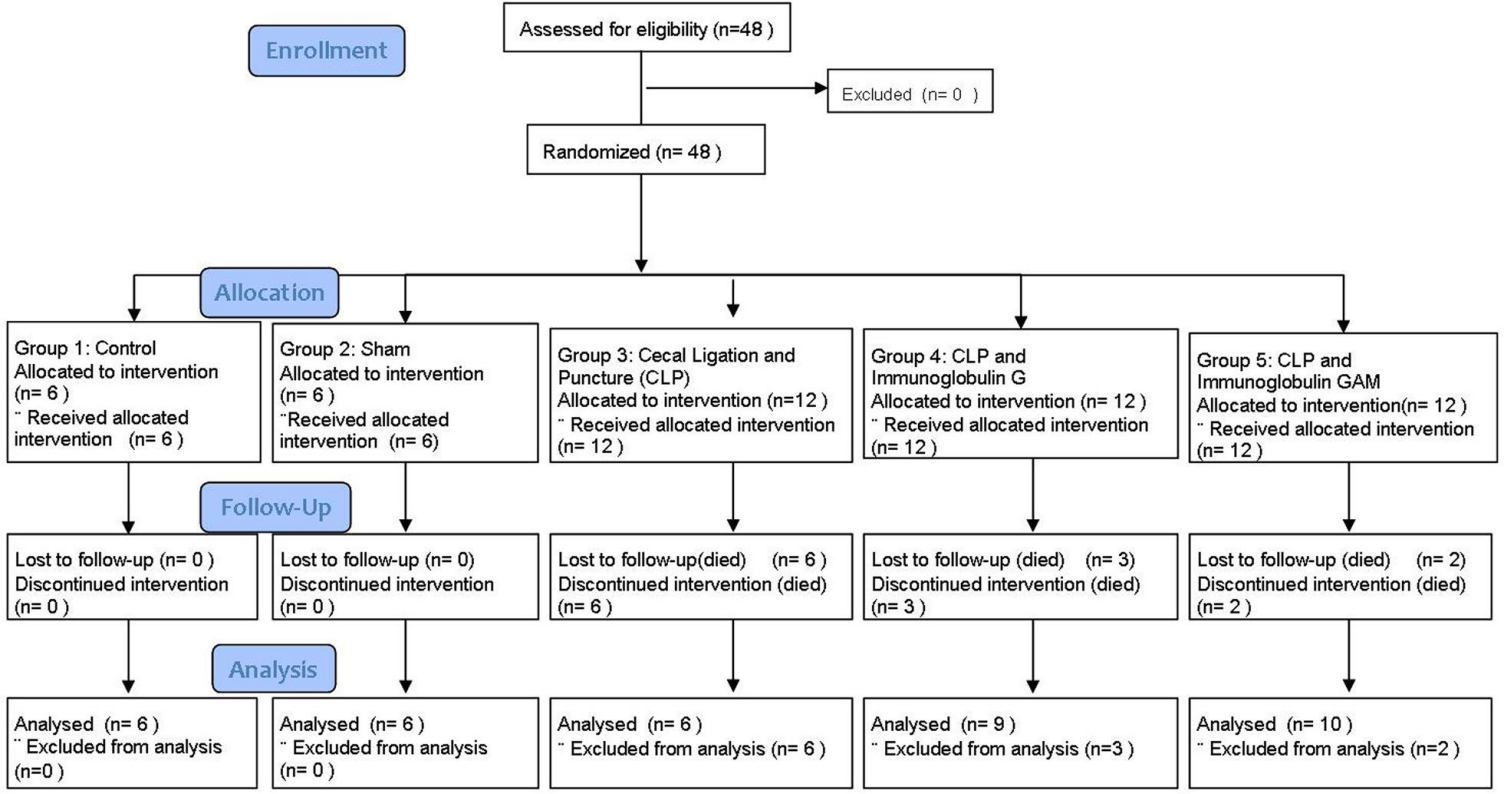
Flowchart of experiment. *CLP* Cecal Ligation Perforation.

We used male Wistar Albino rats (200–250 g). Animals were obtained from Marmara University, Istanbul. Animals were kept in groups under normal conditions (temperature 22 ± 1°C, humidity 55 ± 5%) with access to food and water ad libitum. The procedures of the study were approved by the Local Ethics Committee for Animal Experimentation (78/2012 March). Experimental groups were assigned as control (*n* = 6), sham (*n* = 6), CLP (*n* = 12), IgG (*n* = 12) and IgGAM (*n* = 12). The total sample of 48 subjects achieves 93% power to detect a non-zero contrast of the means versus the alternative that the contrast is zero using an F test with a 0.050 significance level. The value of the contrast of the means is −12.00. The common standard deviation within a group is assumed to be 3.00. Random number table has been used for selection of subject. The randomization was done using online software (http://www.randomization.it) to generate a random allocation sequence. Flowchart is shown in Fig. 1.

### Cecal ligation and puncture procedure

Cecal ligation and puncture procedure was performed as explained by Rittirsch et al. [10]. Animals were intraperitoneally anesthetized with ketamine (100 mg/kg). Rats were placed in a supine position and the abdominal area was shaved. The area was disinfected with alcohol; an incision was made in abdominal wall. The cecum was isolated with anatomical forceps and mesenteric blood vessels were carefully taken care of to prevent dissection. Sepsis was induced with cecal ligation and two punctures were made with an 18-gauge needle through the cecum. A small amount of feces was extruded after removing the needle, and the cecum was replaced into the abdominal cavity. The animals were then treated with pre-warmed saline (37°C; 5 mL per 100 g body weight) subcutaneously, and then moved back into their cages. Sham-operated rats underwent the same procedure, without ligation and puncture of the cecum. After the procedure, antibiotic treatment, clindamycin (150 mg/kg) and ceftriaxone (50 mg/kg) were given intraperitoneally every 6 h for a total 3 days.

### Administration of immunoglobulins

The animals were given human IgG; 250 mg/kg (Octapharma; Vienna, Austria) or IgGAM, 250 mg/kg (Pentaglobin; Biotest, Dreieich, Germany) intravenously via penile vein 5 min after CLP procedure. After the IV injection, the animals were placed back to their cages for recovery. Three behavioral tasks were applied to all animals 10, 30 and 60 days after the surgery.

### Behavioral procedures

#### Open field test

With open field test (OFT), we tried to evaluate the behavior and function in terms of both locomotor activity and daily habits. The OFT was performed on the 10th, 30th and 60th days. In brief, the open field apparatus consists of a rectangular area of 80 × 60 cm surrounded by a 30-cm-high wall. It was divided into 35 equal sized squares with white lines on the floor. A single rat was placed in the center of the area and observed for 5 min. The number of center squares passed, total squares passed, time spent in the center squares, time spent in the four corner squares, numbers of rearing and grooming events, and the number of defecations were recorded. The apparatus was thoroughly cleaned using 70% alcohol after each testing period. The scores were computed for further statistical analysis [11].

#### Elevated plus maze

The elevated plus maze (EPM) was performed on the 10th, 30th and 60th days. The EPM apparatus consisted of two opposite open arms (42.5 cm × 14.5 cm), and two opposite arms of equal size enclosed by walls 30 cm in height, with open tops. The arms were connected by a central 10 × 10 cm square, giving the maze the shape of a plus sign. The maze was elevated 78.5 cm from the floor. All rats were placed individually in the center of the maze facing a closed arm and allowed 5 min of free exploration. Entries and time spent in open and closed arms were measured. The EPM was thoroughly cleaned using 70% ethanol after each rat. The scores were computed for further statistical analysis [12].

#### Forced swim test

The forced swim test (FST) was performed at the end of the study. It was conducted by placing all rats individually in the testing cylinders (45 cm high × 20 cm in diameter) filled with 30 cm water, maintained at 25 ± 1°C. The rats were allowed to swim in the cylinder under conditions in which escape is impossible. In the first trial, rats were allowed to swim for 12 min. They were then removed from water, dried and put into a warmed place for approximately 15 min. After 24 h, the second trial was performed. This trial lasted 5 min and was recorded by a video camera. In this trial, we measured duration of immobility, which is explained as the absence of motion of the body except for small action necessary to keep the rat’s head above the water. After 5 min, rats were taken from the cylinder, dried and placed in a warmed place for approximately 15 min before returning to home cages. After the procedure, the devices were emptied and cleaned following every session [13].

### Statistical analysis

Results from the tests are presented as mean ± SEM. Data were analyzed by one-way analysis of variance (ANOVA) followed by the Tukey’s multiple comparison test, using GraphPad Prism 5. Statistical significance was set as *p* < 0.05.

## Results

Overall mortality rate found 50% in septic animals was dropped to 30 and 20% by IgG and IgGAM, respectively. The number of rats which behavioral tasks have been applied at 10th, 30th and 60th days’ time points were 6, 4, 4 in CLP group, 11, 9, 9 in IgG group and 11, 10, 10 in IgGAM group, respectively. The results of the behavioral alterations caused by CLP-induced sepsis assessed by open field, elevated plus maze and forced swimming tasks are shown as follows:

### Open field test

**Fig. 2:**
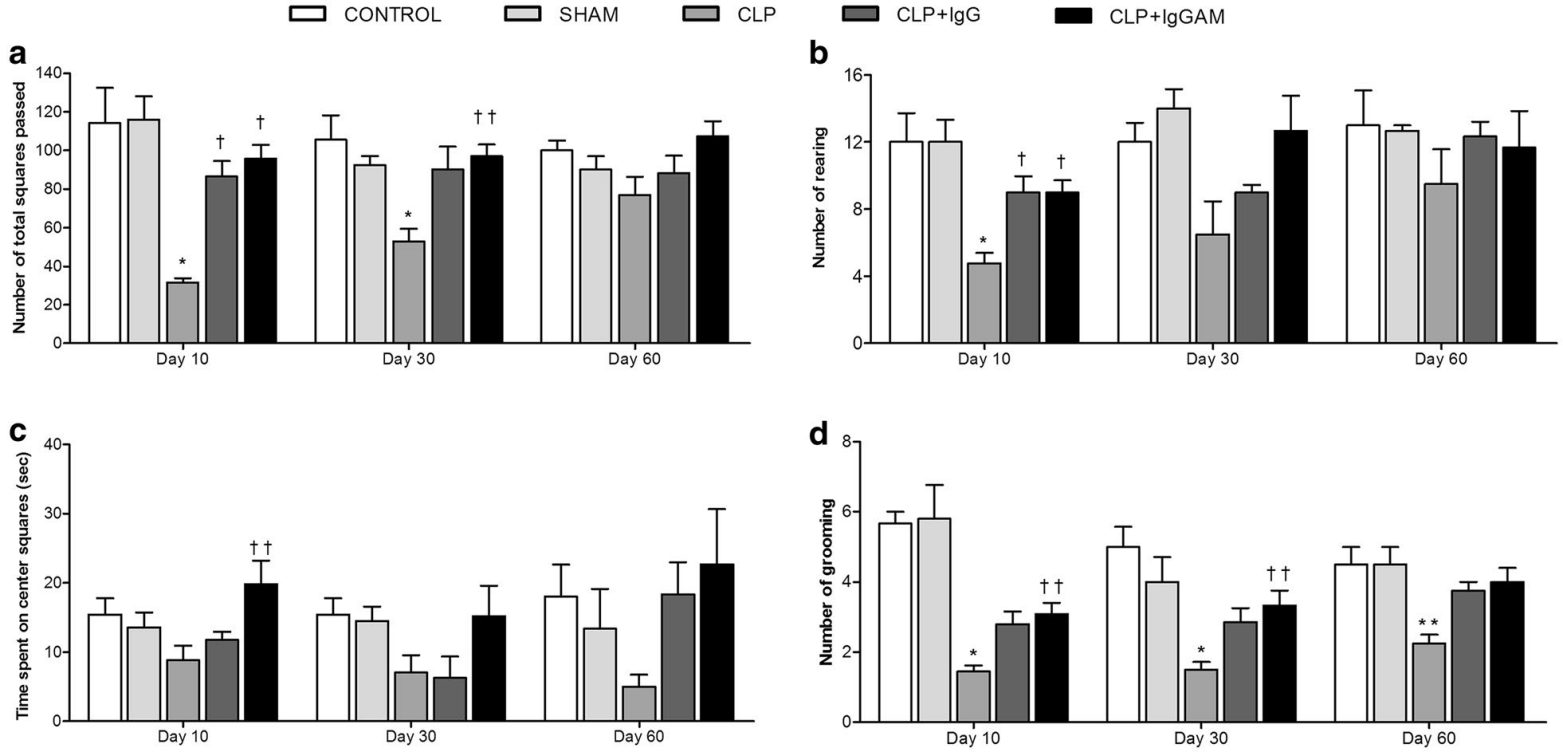
Open field test on days 10, 30 and 60. **a** Number of squares passed in OFT. **b** Number of total rearing movements in OFT. **c** Time spent on center squares in OFT. **d** Number of grooming bouts in OFT. Data are shown as mean ± SEM; *p < 0.01 compared to control, **p < 0.05 compared to control, ^†^ *p* < 0.01 compared to CLP, ^††^ *p* < 0.05 compared to CLP. *OFT* open field test, *CLP* Cecal Ligation Perforation.

The number of crossed squares was not statistically different between sham and control groups. The values of OFT decreased in the CLP group compared with controls on day 10 and 30 (Fig. 2a; *p* < 0.01). When compared with the CLP group, the number of crossed squares increased on day 10 and 30 in the IgGAM group (*p* < 0.01, *p* < 0.05, respectively), whereas it increased only on day 10 in the IgG group (*p* < 0.01) but not on day 30. There was no significant difference on day 60 among the groups (Fig. 2a). The number of rearing events reduced in the CLP group compared with control values on day 10 (*p* < 0.01) but not on day 30 or 60 (Fig. 2b). Both immunoglobulin treatments were able to elevate the number of rearing events compared with CLP group only on day 10 (Fig. 2b, *p* < 0.01). Overall, there was no statistically difference between control and sham groups.

With the exception of the IgGAM group who spent more time in the central zone than the CLP group on day 10, there was no statistical difference in this parameter among all groups/measurements (Fig. 2c, *p* < 0.05). Grooming events significantly decreased in the CLP group compared with controls on day 10 (*p* < 0.01), 30 (*p* < 0.01) and 60 (Fig. 2d, *p* < 0.05). The IgGAM group showed higher grooming activity than the CLP group on day 10 and 30 (Fig. 2d, *p* < 0.05). The IgG group was not statistically different from CLP or other groups. Overall there was no statistical difference between the control and sham groups (Fig. 2d).

### Elevated plus maze

**Fig. 3:**
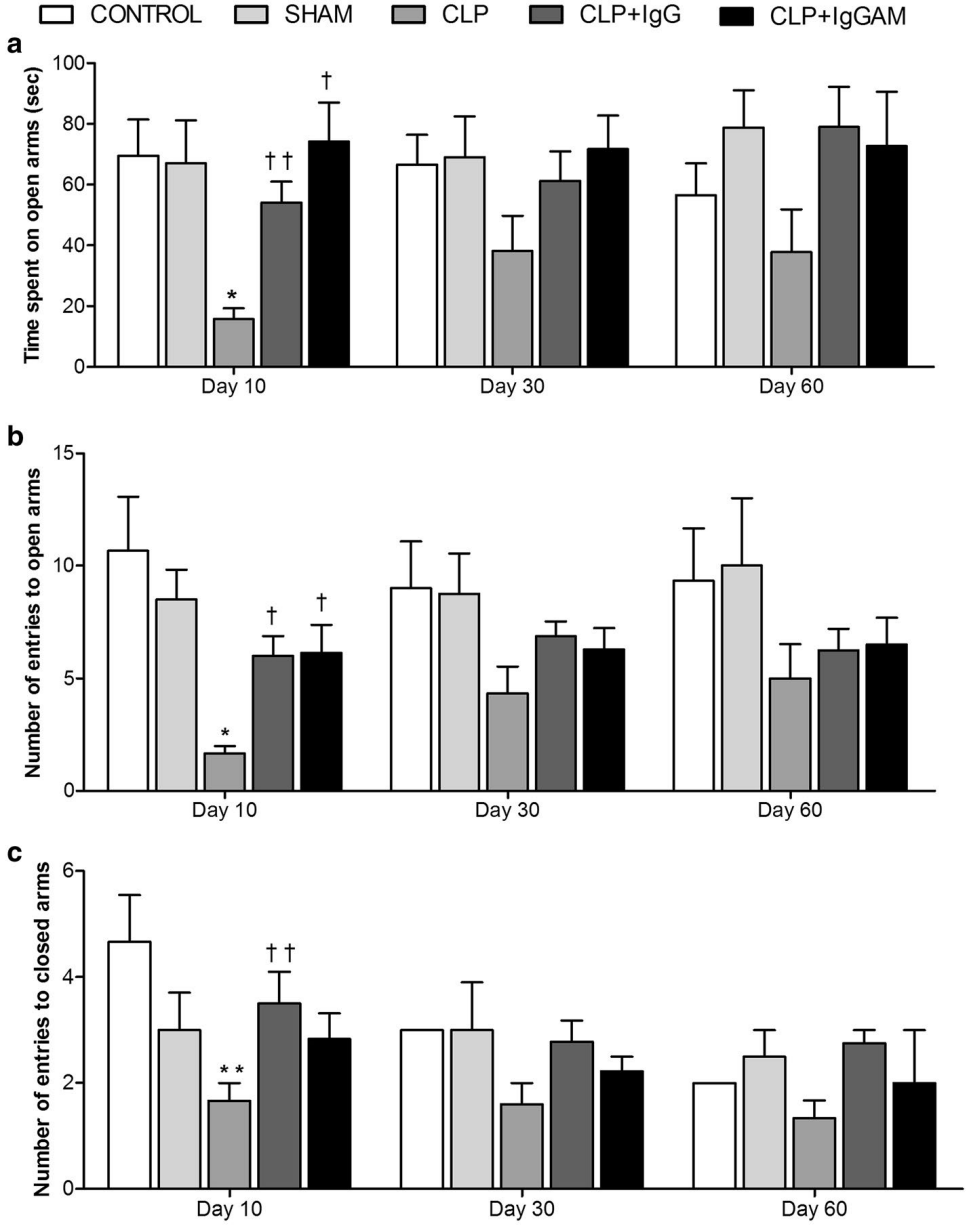
Elevated plus maze test on days 10, 30 and 60. **a** Time spent on open arms in EPM test. **b** Number of entries to open arms in EPM test. **c** Number of entries to closed arms in EPM test. Data are shown as mean ± SEM; *p < 0.01 compared to control, **p < 0.05 compared to control, ^†^ *p* < 0.01 compared to CLP. ^††^ *p* < 0.05 compared to CLP. *EPM* elevated plus maze; CLP, Cecal Ligation Perforation.

Anxiety-like behavior is evaluated with EPM. Time spent on open arms was decreased in the CLP group when compared with controls on day 10 (*p* < 0.01). IgG and IgGAM treatments increased the time spent on open arms compared with the CLP group on day 10 (Fig. 3a, *p* < 0.05, *p* < 0.01, respectively). There was no significant difference in time spent on open arms between the groups on day 30 and 60 (Fig. 3a). The number of open and closed arms entries were significantly decreased in the CLP group compared with controls on day 10 (Fig. 3b, c, *p* < 0.01 and *p* < 0.05, respectively), whereas both the IgG and IgGAM treatments showed a higher number of entries to open arms compared with the CLP group on day 10 (Fig. 3b, *p* < 0.01). Closed arm entries were only higher in the IgG group when compared with the CLP group (Fig. 3c, *p* < 0.05) on day 10. All groups showed similar results on day 30 and 60 (Fig. 3b, c).

### Forced swim test

**Fig. 4:**
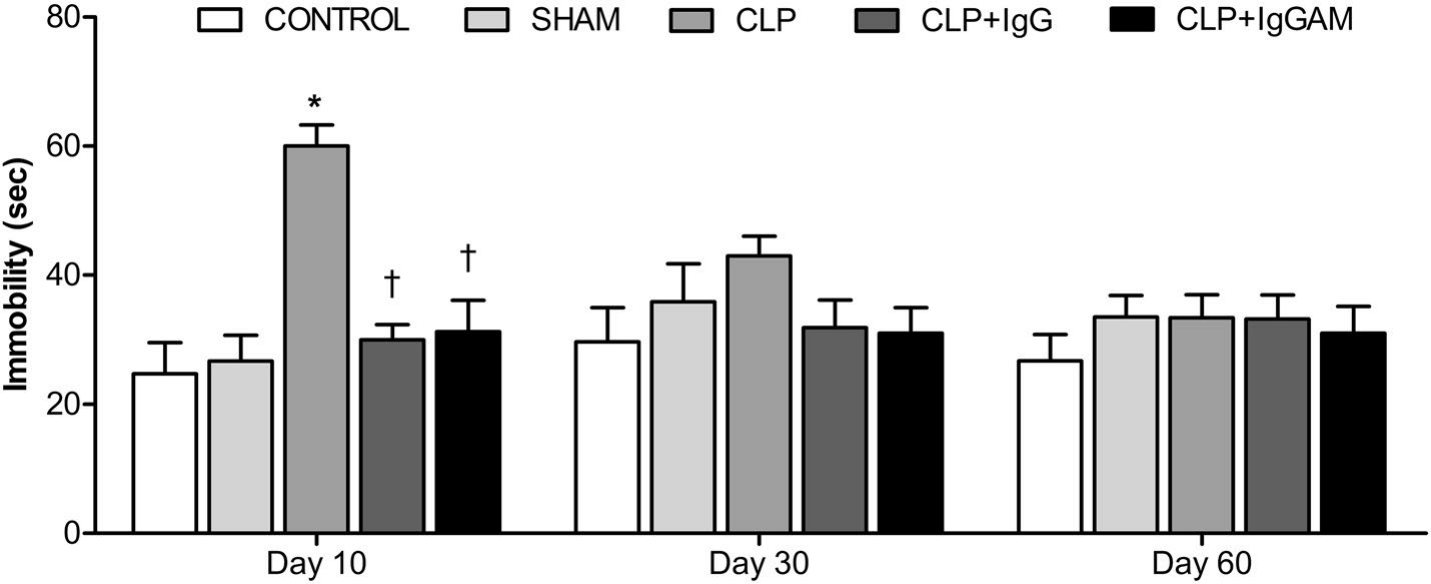
Forced swimming test on days 10, 30 and 60. Immobility times in FST. Data are shown as mean ± SEM; *p < 0.01 compared to control, ^†^ *p* < 0.01 compared to CLP. *FST* forced swimming test, *CLP* Cecal Ligation Perforation.

Depressive-like behavior is evaluated with FST. The time of immobility was greater in the CLP group compared with the control group on day 10 (Fig. 4, *p* < 0.01). On the other hand, both IgG and IgGAM treatments showed decreased immobility time compared with the CLP group on day 10 (Fig. 4, *p* < 0.01). There was no significant difference in immobility time among the groups on day 30 and 60 (Fig. 4).

## Discussion

The present study demonstrated that sepsis induced by CLP resulted in behavioral deficits in open field, elevated plus maze and forced swimming tasks 10 days after surgery that mainly resolved within 30 days. The main finding of the study is that treatment with immunoglobulin after sepsis induction caused earlier resolution in locomotor function and behavioral alterations including depression and anxiety. All surviving animals presented nearly normal performance in all tests performed after 60 days indicating recovery of functional disturbances. The overall survival rate in the CLP group was 50% in accordance with other studies, whereas it was 70% after the standard IgG and 80% in the Ig GAM treatment groups.

Long-term cognitive impairment including alterations in memory, anxiety, depression and impairment in executive function was reported in sepsis survivors [14]. Each of these was shown to be associated with a decreased quality of life. CLP model was extensively used to address the mechanisms associated with cognitive deficits seen in sepsis and help to investigate therapeutic approaches for this problem [3, 15]. Among cognitive functions, memory was reported to be the most frequently observed deficit. The present study evaluated the changes in locomotor activity, anxiety and depressive-like symptoms in sepsis-surviving rats.

We studied three basic behavioral tasks that were mainly used in those experiments determining long-term effects in sepsis-surviving animals. Forced swimming test was used as a model for determining depressive-like behavior [13], elevated plus maze test was used for anxiety measurement [12], and animals were put into different open field facilities to determine the alterations in locomotor and functional activities [11]. Time-dependent measurements in CLP-induced septic animals were in accordance with previous reports in terms of decreases in locomotor activity and increases in depressive-like behavior [8]. Rearing and grooming events dramatically decreased in animals in the CLP group, which indicated attenuation in the locomotor activity by the induction of sepsis. The activity was attenuated on day 10 and 30. These results were in contrast with the previous reports, which indicated no change in the locomotor and exploratory activities in the open field test in the CLP group compared with control group, 10 days after the procedure [3, 8, 15–17]. Similar to these, Leite et al. found no significant effects of CLP on the locomotor activity in OFT 7 days after surgery compared with the sham group [18]. Overall, both immunoglobulin treatments were able to elevate the number of rearing and grooming events compared with the CLP group on day 10, whereas the IgGAM group was also able to increase grooming on day 30. Although there was no difference in terms of the time spent in the central zone when the CLP group compared with the control group, the IgGAM group stays longer than CLP group in the central zone on day 10, which indicated a better reversal of anxiety-like behavior.

In the FST, the time of immobility, indicating a depressive-like behavior, was higher in the CLP group compared with the control group on day 10, which is compatible with previous reports that used the CLP model. Two previous studies reported a significant increase in the immobility time in the CLP group compared with the sham group, 10 days after CLP procedure [5, 15]. In addition, a recent study that used a sucrose consumption test as a depressive-like state model reported that CLP induction caused anhedonia with the reduced sucrose intake compared with the sham group, 17 days after surgery [17]. We found no differences in the time of immobility between the groups on day 30 and 60 meaning that depressive-like state was resolved before 30 days in the CLP group. In a previous study, the CLP group was shown to have increased immobility time compared with the sham group on day 10 and day 30, but not on day 60 [8]. We showed that both IgG and IgGAM treatments decreased immobility time compared with the CLP group on day 10, which can be interpreted that both treatments reversed CLP-induced depressive-like behaviors by day 10.

The CLP procedure produced an anxiogenic-like effect 10 days after surgery. This was demonstrated by the decreased time spent in open and closed arms of the EPM task. This was not the case in the previous experimental trials that studied long-term depression and anxiety after sepsis. Two experimental trials demonstrated no change in the anxiety-like behavior after CLP [3, 5]. Most recently, Leite et al. demonstrated significant anxiety in CLP-induced septic animals, which is in accordance with our findings [18]. In the present study, anxiety-like behavior had resolved in the CLP group by day 30 after surgery. Both immunoglobulin treatments increased the time spent in open arms, which was coupled with higher number of entries to open arms compared with the CLP group on day 10. Treatment with IgG and IgGAM caused early resolution of functional and behavioral alterations after sepsis induction, which indicated some treatment effect. This was parallel to the improved survival in the treatment groups. The underlying mechanisms of which immunoglobulin exerts these effects are most probably due to their regulatory involvement in the inflammatory processes causing endothelial activation and the breakdown of the BBB [19]. In our previous experimental trial, immunoglobulin treatment protected the brain from sepsis-induced effects [9]. Fewer morphologic changes and less disruption in BBB integrity were evident, which was in accordance with the improved survival in the treatment groups. The BBB failure in sepsis is explained by endothelial cell injury through cytokines such as TNF-α and IL-β that leads to upregulation of endothelial surface antigens and subsequent white blood cells adherence as well as microcirculatory dysfunction [20]. Experimental studies have shown that IVIG is capable of significantly reducing leukocyte adhesion and by normalizing capillary perfusion attenuate microcirculatory dysfunction [21]. Using intravital microscopy, the effects of immunoglobulins on leukocyte recruitment in superficial brain vessels were visualized. Immunoglobulins potentially reduced leukocyte rolling and adhesion in experimental autoimmune encephalomyelitis (EAE) [22]. It has been suggested that immunoglobulins exert these actions either through their effects on cytokine production or directly by reducing cell adhesion molecule production [22, 23]. Experiments using cultures of bovine and human brain endothelium suggest that cytokines increase BBB permeability. TNF-α was stained on microvascular vessels of the brain in sepsis and recombinant TNF-α also increased the permeability in mice [20]. Studies demonstrated that immunoglobulins contain high-affinity neutralizing antibodies against IL-1 IL-6 and TNF-α and downregulate their synthesis by their effects on T cells [21, 24].

The beneficial effects of IV immunoglobulins on the BBB are most probably due to their regulating effects on the complement system [25]. The neurotoxic effects of complement fragments have recently been studied and blocking C5a anaphylatoxin resulted in neuroprotective effects in CLP-induced septic animals [26]. Arumugam et al. have demonstrated that complement activation in neuroinflammation was regulated with immunoglobulins in an experimental stroke model, and immunoglobulins reduced the mortality and the amount of brain damage [27].

In the current study, two different types of immunoglobulin preparations were used expecting that IgM would improve functional and behavioral alteration caused by sepsis. *Early experimental* studies have demonstrated superior effects of IgM in comparison with IgG in terms of their activities on inflammation [28, 29]. Both intravenous IgM and IgG preparations markedly attenuated the endotoxin-induced leukocyte adherence; however, only intravenous IgM was capable of further reducing venular leukocyte adherence, whereas IgG did not show protective effects compared with controls. This effect was also evident with the measurement of functional capillary density (FCD) where IgM significantly ameliorated the LPS-induced decrease of FCD after 24 h of endotoxemia [21]. Protective effects of IgM on tissue integrity were studied on lungs where significantly reduced alveolar damage was evident parallel with the histological evaluation [30]. This study showed a better survival rate with IgM-enriched immunoglobulin administration during sepsis; however, both immunoglobulin preparations were able to reverse behavioral deficits within 10 days, which were resolved by 60 days after surgery in all groups.

One limitation of the study might be considered as the possible cross-species binding differences since we used human IV immunoglobulin preparation in rats as rodents that are routinely used as a convenient first-line model for clinical evaluation of IVIG therapies. Studies on the mapping of the binding side on human immunoglobulins suggested that the Fc interactions are, in some respects, very similar across species [31]. Second, the timing of IVIG administration might be considered as too early as to extrapolate its impact for clinical effectiveness; however, similar timing of IVIG administration has been widely used in experimental studies testing the potential of therapeutic effects. Third, in our setup, we were not able to evaluate a memory deficit which was reported to be the most frequently observed deficit in humans.

## Conclusions

In the present experimental study, we show that that intravenous immunoglobulins provide early behavioral and functional recovery following sepsis. Our data are the first to show a better resolution of effects with immunoglobulin in sepsis-induced behavioral alterations. Further researches are necessary to see if our findings can be extrapolated to humans.

## Abbreviations

IgG: immunoglobulin G
IgA: immunoglobulin A
IgGAM: immunoglobulins enriched with IgA and IgM
CLP: cecal ligation and puncture
BBB: blood–brain barrier
OFT: open field test
EPM: elevated plus maze
FST: forced swim test
ANOVA: one-way analysis of variance
